# Persistent left superior vena cava: a case report and review of literature

**DOI:** 10.1186/1476-7120-6-50

**Published:** 2008-10-10

**Authors:** Sandeep K Goyal, Sujeeth R Punnam, Gita Verma, Frederick L Ruberg

**Affiliations:** 1 Department of Medicine, Boston University School of Medicine, Boston, MA, USA; 2 Department of Medicine, Section of Cardiology, Boston University School of Medicine, Boston, MA, USA; 3 Department of Radiology, Boston University School of Medicine, Boston, MA, USA; 4 Division of Cardiology, Michigan State University, East Lansing, MI, USA; 5 Maulana Azad Medical College, New Delhi, India

## Abstract

Persistent left superior vena cava is rare but important congenital vascular anomaly. It results when the left superior cardinal vein caudal to the innominate vein fails to regress. It is most commonly observed in isolation but can be associated with other cardiovascular abnormalities including atrial septal defect, bicuspid aortic valve, coarctation of aorta, coronary sinus ostial atresia, and cor triatriatum. The presence of PLSVC can render access to the right side of heart challenging via the left subclavian approach, which is a common site of access utilized when placing pacemakers and Swan-Ganz catheters. Incidental notation of a dilated coronary sinus on echocardiography should raise the suspicion of PLSVC. The diagnosis should be confirmed by saline contrast echocardiography.

## Background

Persistent left superior vena cava (PLSVC) is an uncommon vascular anomaly; however, it is the most common congenital anomaly of thoracic venous system. It is usually asymptomatic and is detected when cardiovascular imaging is performed for unrelated reasons. When a left subclavian approach is used for vascular access, its presence can complicate catheter placement within the right side of heart. Here we present a case that highlights the practical implications PLSVC. Further, we review a diagnostic approach and provide insight into the embryonic basis of this anomaly.

## Case presentation

**Figure 1:**
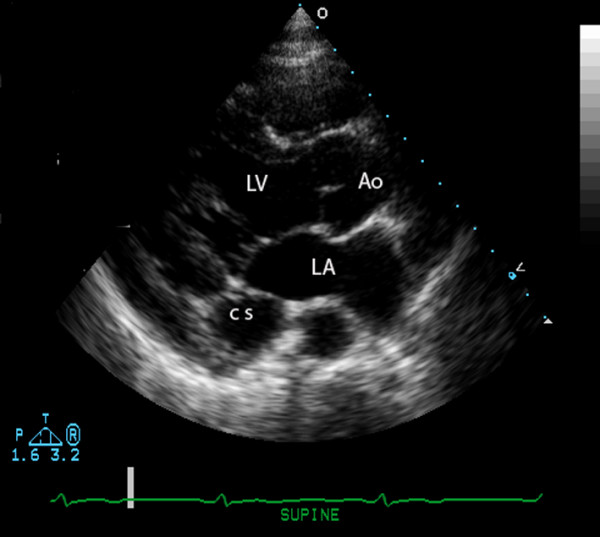
**Transthoracic echocardiogram, parasternal long axis view, illustrating a dilated coronary sinus (CS).** Other chambers visible are the left ventricle (LV), aorta (Ao), and left atrium (LA).

**Figure 2:**
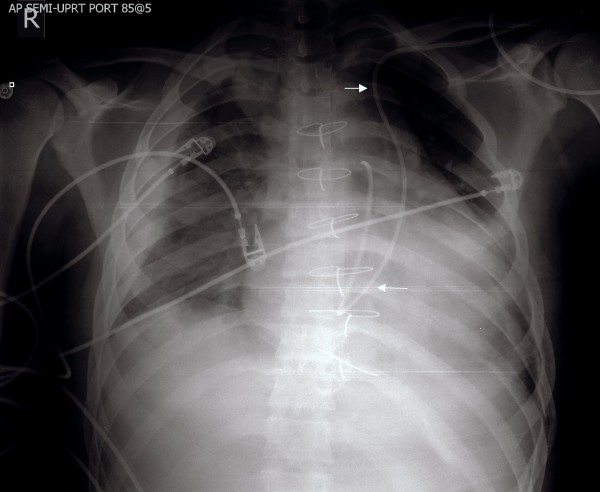
**Chest-x-ray demonstrating unusual course of Swan-Ganz catheter (arrows) with its distal end in the proximal pulmonary artery.** Note the course of the catheter into the heart on the left side of the spine, rather than the right side via the normal anatomic position of the superior vena cava.

**Figure 3:**
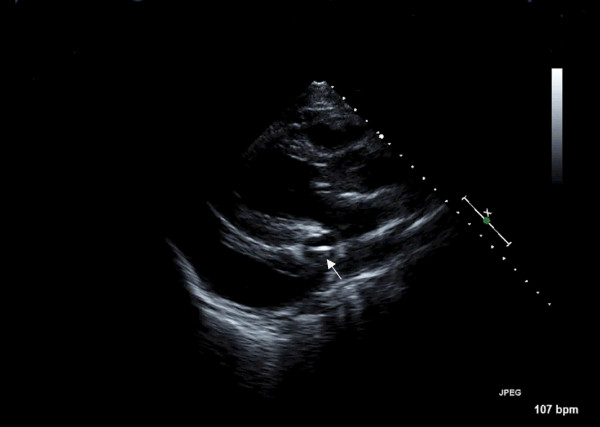
Echocardiogram demonstrating the Swan-Ganz catheter (seen in cross section and indicated by the small arrow) passing via dilated coronary sinus.

**Figure 4:**
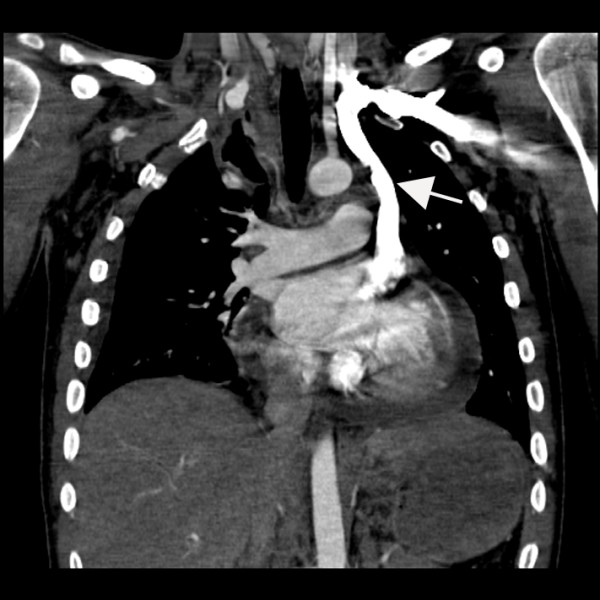
CT thorax showing left sided superior vena Cava (large arrow).

A 19-year-old male was admitted to the hospital after sustaining multiple injuries, including sternal fracture, in a motor vehicle accident. An admission echocardiogram revealed a dilated coronary sinus (Figure 1) with normal right sided filling pressures, raising the suspicion for the presence of a persistent left sided superior vena cava (PLSVC). A follow-up agitated saline ("bubble") study was recommended but not immediately performed. A Swan-Ganz catheter was subsequently placed via the left subclavian approach, and on routine post-procedural chest X-ray, an unusual course of the catheter was identified (Figure 2). PLSVC was suspected, and a follow-up echocardiogram demonstrated the catheter passing through the coronary sinus (Figure 3) (See additional file 1). A Contrast enhanced computed tomography study of thorax was performed to assess the bony and vascular injuries associated with motor vehicle accident, which also revealed PLSVC as an incidental finding (Figure 4). As, the diagnosis was well established on an echocardiogram showing catheter traversing the coronary sinus, a saline contrast echocardiography was deemed unnecessary and was not performed.

## Discussion

Persistent left SVC is the most common congenital thoracic venous anomaly with a prevalence of 0.3–0.5% in general population [1]. The thoracic embryonic venous system is composed of two large veins (the superior cardinal veins) which return blood from cranial aspect of embryo, and the inferior cardinal vein, which returns blood from the caudal aspect. Both pairs of veins join to form right and left common cardinal veins before entering the embryological heart. The left common cardinal vein persists to form coronary sinus and oblique vein of left atrium. During the 8^th ^week of gestation, an anastomosis forms between right and left superior cardinal veins resulting in the innominate (or brachiocephalic) vein. The cephalic portion of superior cardinal veins form the internal jugular veins. The caudal portion of right superior vein forms the normal right-sided superior vena cava, while the portion of the left superior cardinal vein caudal to the innominate vein normally regresses to become "ligament of Marshall". If this normal regression of the left superior cardinal vein fails to occur, a persistent left-sided vascular structure that empties into the coronary sinus, results (the PLSVC). The innominate vein may or may not degenerate in these cases leading to variations in anatomy.

The most common subtype of PLSVC results in the presence of both left and right SVCs. A bridging innominate vein may or may not be present. Webb *et al *[2] reported that a PLSVC is associated with absence of the innominate vein in 65% cases. More rarely, the caudal right superior cardinal vein regresses leading to an absent right SVC with PLSVC. In this case, the left SVC returns all the blood from cranial aspect of the body. Variations have also been reported in the insertion of left SVC. In 80–90% of individuals, the persistent LSVC drains into the right atrium via the coronary sinus and is of no hemodynamic consequence. In the remaining cases, it may drain in left atrium resulting in a right to left sided shunt.

Diagnosis of left SVC is usually made as an incidental finding during cardiovascular imaging or surgery. Placement of Swan-Ganz catheter via the left subclavian approach as in this case of PLSVC demonstrates an unusual course of the catheter on chest X-ray [Fig 1]. Transthoracic echocardiography reveals a dilated coronary sinus and diagnosis can be confirmed by use of saline contrast ("bubble study") echocardiography. PLSVC is not the only cause of a dilated coronary sinus, however, with other etiologies including elevated right atrial pressure (most common), coronary arterio-venous fistula, partial anomalous pulmonary venous return, or an "unroofed" coronary sinus affording shunt flow between the left atrium and coronary sinus. The following diagnostic criteria can be used with echocardiography: (1) the presence of a dilated coronary sinus on two-dimensional echocardiography in the absence of evidence of elevated right sided filling pressures; (2) enhancement of the dilated coronary sinus before the right atrium (RA) after contrast material infusion into a left arm vein; (3) normal transit of contrast with RA opacification before the coronary sinus with contrast injected from the right arm. Multislice computed tomography [Fig 4] or magnetic resonance venography can also be employed to establish the diagnosis, and is useful to rule out variations in the typical anomalous venous course. Single or multiplane transesophageal echocardiography [3] and radionuclide angiocardiography have also been used to establish diagnosis.

Almost 40% of patients with PLSVC can have a variety of associated cardiac anomalies, [4,5] such as atrial septal defect, bicuspid aortic valve, coarctation of aorta, coronary sinus ostial atresia, and cor triatriatum. The presence of associated anomalies is more common with concomitant absence of right SVC the notation of which warrants appropriate investigation to rule out other anomalies. The PLSVC has been associated with anatomical and architectural abnormalities of the sinus node and conduction tissues. Both sinus and AV node can have persistent fetal dispersion in the central fibrous body in subjects with PLSVC[6].

PLSVC has various practical implications when the left subclavian vein is used for access to the right side of the heart or pulmonary vasculature. Swan-Ganz catheter placement can be challenging as it is performed without imaging under many circumstances, such as at the bedside. PLSVC can also complicate permanent pacemaker and implantable cardioverter defibrillator (ICD) placement (the latter of which is always done under fluoroscopic guidance thus the anomaly is typically detected during the procedure). Serious complications such as arrhythmia, cardiogenic shock, cardiac tamponade, and coronary sinus thrombosis have been reported when pacemaker leads or catheters have been inserted via PLSVC. Fortunately, the incidence of such complications is relatively low, and permanent pacemaker leads for single chamber pacing have been successfully placed via PLSVC as early as 1971 [7].

Improvements in catheter types and technique over time have permitted the successful placement of right atrial and right ventricular leads for dual-chamber pacing [8]. In addition, cardiac resynchronization therapy for advanced chronic heart failure requires the placement of third pacing lead in left posterolateral vein of the heart. Several operators have successfully placed a cardiac resynchronization system, and therefore a lead via the coronary sinus, in individuals with PLSVC with good intermediate term results [9].

During cardiac surgery, the presence of PLSVC is a relative contraindication to the administration of retrograde cardioplegia. It may be possible to clamp the PLSVC to prevent the cardioplegia solution from perfusing retrograde up the PLSVC and its branches with inadequate myocardial protection [10]. However, there is a possibility that there may be some steal of cardioplegia solution through an accessory vein. During heart transplantation in a patient with PLSVC, the coronary sinus must be dissected carefully to permit reanastomosis of PLSVC to right atrium[11].

## Conclusion

Presence of a dilated coronary sinus on echocardiography should alert the clinician towards the possibility of PLSVC. The diagnosis should be confirmed by saline contrast echocardiography. Cardiologists and critical care physicians should consider presence of PLSVC whenever a catheter or guide wire inserted via left subclavian vein takes an unusual left-sided downward course. A PLSVC certainly presents technical difficulties with right heart access via the left subclavian, but does not preclude insertion of catheters; however, the additional associated risks should be discussed with the patient if the diagnosis of PLSVC is already established, and alternative access sites should be considered.

## Consent

Written informed consent was obtained from the patient for publication of this case report and accompanying images. A copy of the written consent is available for review by the Editor-in-Chief of this journal.

## Competing interests

The authors declare that they have no competing interests.

## Authors' contributions

SKG collection of data, patient care and preparation of manuscript. SRP, GV preparation of manuscript. FLR final revision of manuscript and guidance. All authors read and approved the final manuscript.

## Supplementary Material

Additional file 1 **Echocardiographic movie of parasternal long axis view showing Swan-Ganz catheter traversing the dilated coronary sinus.** Click here for file
